# Store-Operated Ca^2+^ Entry (SOCE) Contributes to Normal Skeletal Muscle Contractility in young but not in aged skeletal muscle

**DOI:** 10.18632/aging.100335

**Published:** 2011-06-06

**Authors:** Angela M Thornton, Xiaoli Zhao, Noah Weisleder, Leticia S. Brotto, Sylvain Bougoin, Thomas M. Nosek, Michael Reid, Brian Hardin, Zui Pan, Jianjie Ma, Jerome Parness, Marco Brotto

**Affiliations:** ^1^ Department of Physiology & Biophysics, Robert Wood Johnson Medical School and; ^2^ Rutgers University Physiology and Integrative Biology and Department of Biomedical Engineering, Piscataway, New Jersey, 08854; ^3^ The Muscle Biology Research Group-MUBIG, Schools of Nursing and Medicine, University of Missouri-Kansas City, Missouri, Kansas City, Missouri, 64108; ^4^ Departments of Anesthesiology and Pharmacology and Chemical Biology, University of Pittsburgh School of Medicine and Children's Hospital of Pittsburgh, Pittsburgh, Pennsylvania, 15122; ^5^ Department of Physiology & Biophysics, Case Western Reserve University, Cleveland, Ohio, 44106; ^6^ Department of Physiology & Biophysics, University of Kentucky, Lexington, Kentucky, 40504; ^+^ In Memoriam of Brian Hardin

**Keywords:** SOCE, calcium entry, muscle contraction, muscle aging, aging

## Abstract

Muscle atrophy alone is insufficient to explain the significant decline in contractile force of skeletal muscle during normal aging. One contributing factor to decreased contractile force in aging skeletal muscle could be compromised excitation-contraction (E-C) coupling, without sufficient available Ca^2+^ to allow for repetitive muscle contractility, skeletal muscles naturally become weaker. Using biophysical approaches, we previously showed that store-operated Ca^2+^ entry (SOCE) is compromised in aged skeletal muscle but not in young ones. While important, a missing component from previous studies is whether or not SOCE function correlates with contractile function during aging. Here we test the contribution of extracellular Ca^2+^ to contractile function of skeletal muscle during aging. First, we demonstrate graded coupling between SR Ca^2+^ release channel-mediated Ca^2+^ release and activation of SOCE. Inhibition of SOCE produced significant reduction of contractile force in young skeletal muscle, particularly at high frequency stimulation, and such effects were completely absent in aged skeletal muscle. Our data indicate that SOCE contributes to the normal physiological contractile response of young healthy skeletal muscle and that defective extracellular Ca^2+^ entry through SOCE contributes to the reduced contractile force characteristic of aged skeletal muscle.

## INTRODUCTION

Aging is a complex biological process marked by the gradual decline of a multitude of physiological processes [1-5]. Some functional changes, such as decreased muscular strength, have a tremendous impact on the quality of life. Normal aging involves sarcopenia, a combination of atrophy and decreased muscular strength that develops despite dietary interventions and increased physical activity [6, 7]. The physical, psychological and socio-economic impact of sarcopenia is largely underestimated despite the fact that it is a leading contributor to debilitating injuries due to repetitive falls, loss of independence, and a reduced quality of life in the elderly population [4]. Understanding the cellular mechanisms that contribute to sarcopenia is essential for the development of effective treatments and improved care for the elderly.

While many cellular modifications [8-13] may contribute to muscle aging, excitation-contraction (E-C) coupling is an elemental process that must be considered because it is so integral to the control of muscle contractility. E-C coupling requires the close coordination of extracellular Ca^2+^ ([Ca^2+^]_o_) entry or voltage-sensing changes with Ca^2+^ release from the principal intracellular storage organelle, the sarcoplasmic reticulum (SR). Changes in E-C coupling machinery may act as causative factors for, or adaptive mechanisms in, muscle aging that directly contribute to muscle weakness. Decreased muscle function during aging has been associated with muscle fiber denervation, loss of motor units, and motor unit remodeling. Moreover, altered function of several triad junction proteins involved in the regulation and transduction of E-C coupling has been shown to contribute to disrupted Ca^2+^ homeostasis in aged skeletal muscle [14-17].

Thus, reduced homeostatic capacity for intracellular Ca^2+^ movement may underlie the progression of sarcopenia and contractile dysfunction during muscle aging. We have previously demonstrated that changes in aged skeletal muscle Ca^2+^ homeostasis are accompanied by distinct modification of the structure and protein composition of triad junctional complexes. We found that expression of mitsugumin 29 (MG29), a triad junction protein, drastically decreases with aging and that skeletal muscle from adolescent MG29 knockout mice contain many of the phenotypic changes we observe in aged skeletal muscle, including defective store-operated Ca^2+^ entry (SOCE) [16-18]. SOCE is an important mechanism linking [Ca^2+^]_o_ entry and intracellular Ca^2+^ storage, in particular during the repetitive cycles of E-C coupling when a reduced SR Ca^2+^ store necessitates the activation of SOCE. Several previous studies have demonstrated that SOCE plays a role in muscle fatigue [19-22] as part of a functional stress response in skeletal muscle [18].

However, to date, to the best of our knowledge, no systematic studies have yet been performed to test if a reduced SOCE function contributes to the decreased contractile capacity of aged skeletal muscles. Furthermore, it is not known if and how SOCE contributes to normal muscle contractility, two essential physiological questions that we aimed to answer in these novel studies.

To examine the contribution of SOCE to muscle contractile function during aging, we made use of *ex vivo* contractility assays, in which the components of the extracellular milieu surrounding isolated anatomical muscles can be precisely manipulated. This allowed use of pharmacological and experimental manipulations to alter the function of SOCE in order to define its physiological role in young and aged skeletal muscle. We found that reagents that prevent [Ca^2+^]_o_ entry reduce contractile force in skeletal muscle, an effect that was more prominent at high frequency stimulation as compared to lower frequencies of stimulation, and that SOCE is a robust mechanism of [Ca^2+^]_o_ entry in young, healthy skeletal muscle. Of utmost importance, we also demonstrate that SOCE is compromised in aged skeletal muscle, and that loss of this physiological component contributes to at least a part of the decreased contractile force generation typical of aged muscle.

Our novel studies that now link SOCE with muscle contractile function, suggest that manipulation of SOCE may present a therapeutically valid target for improvement of contractile function and, potentially the amelioration of muscle weakness seen during aging.

## RESULTS

### Extracellular Ca^2+^ contributes to tetanic contractile force in skeletal muscle

**Figure 1:**
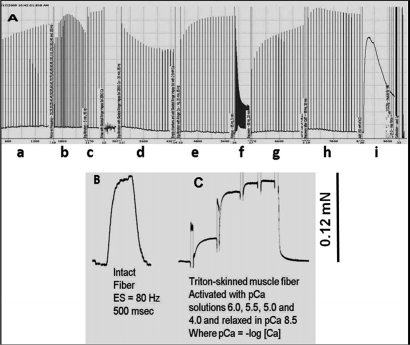
Original and unfiltered recording of a SOL muscle from Wt mice illustrates robustness of our preparations as it can undergo a multitude of experimental manipulations during a prolonged protocol without signs of deterioration. **(A)** An entire protocol of more than 6h in duration in a soleus (SOL) muscle is shown. The experimental points shown in this record are: a) Initial equilibration period where muscle is carefully stretched; b) Force vs. frequency relationship, c) Equilibration, followed by solution change (from 2.5 mM Ca^2+^ to zero Ca^2+^ + 0.1 mM EGTA), d) Equilibration in Zero Ca^2+^, followed by change of solution back to 2.5 mM Ca^2+^, e) Recovery in 2.5 mM Ca^2+^, f) Fatiguing stimulation, g) Recovery from fatigue in the absence of caffeine, h) Recovery in the presence of 20 mM caffeine, i) After stimulation being stopped, the muscle is treated with 80 mM KCl and still produces a very large response to KCl that approximates the maximal tetanic force. Preparation produced 11 g (~107 mN) of relative force. **(B)** A single muscle fiber was dissected from the same intact SOL muscle shown in *panel A*. Single muscle fiber was the electrically stimulated with 80 Hz. and produced ~ 0.12 mN of force. The same fiber was then chemically skinned with Triton X-100 and produced a maximal contractile force of ~ 0.12 mN. From our own observations, we estimate that a SOL muscle has ~925 muscle fibers; thus, the predicted contractile force for this preparation is ~111 mN (0.12 mN/fiber × 925 = 111 mN), which is very similar to the relative force of 107 mN determined by the force calibration in our intact muscle system.

In the first series of *ex vivo* contractility experiments, it was critical to validate that muscle preparation deterioration or run-down did not play any role in our results. Fig. 1 shows an example that is representative of the muscle preparations used in our studies. Fig. 1A demonstrates that our preparations can undergo very lengthy protocols without any detectable run-down of contractile force. When a single muscle fiber is dissected from the intact muscle, it produces a maximal tetanic force that is equivalent to the amount force produced by the same fiber after it is permeabilized with Triton X-100 and maximally activated with calcium (Figs. 1B, C). These results illustrate the robustness of our preparations and the validity of our force calibrations.

**Figure 2:**
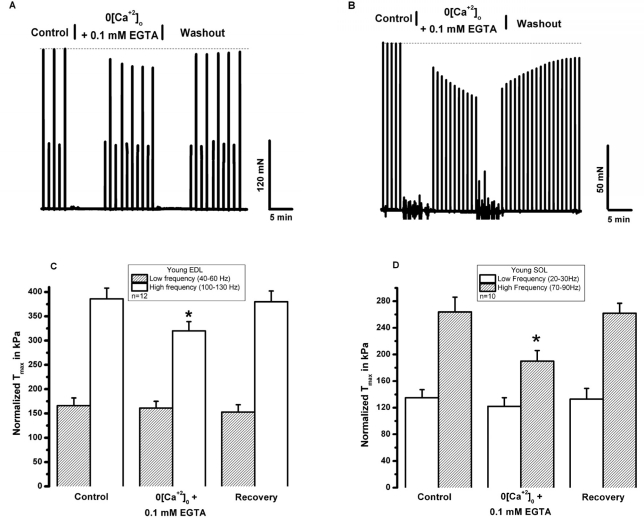
Removal of [Ca^2+^]_o_ reduces tetanic contractile force in young skeletal muscle. **(A)** An Intact EDL muscle was electrically stimulated with low and high frequency in a bath solution with either 2.5 mM [Ca]_o_ (control) or 0 [Ca]_o_+0.1 mM EGTA. **(B)** An Intact SOL muscle was electrically stimulated with high frequency in a bath solution with either 2.5 mM [Ca]_o_ (control) or 0 [Ca]_o_+0.1 mM EGTA. Fast recovery upon return of preparations to 2.5 mM [Ca]_o_ is observed. **(C)** Data summary for the effects of 0 [Ca]_o_ on EDL muscle at T = 25° C (n = 12, p < 0.01). **(D)** Data summary for the effects of 0 [Ca]_o_ on SOL muscle at T = 25° C (n = 10, p < 0.01).

**Figure 3:**
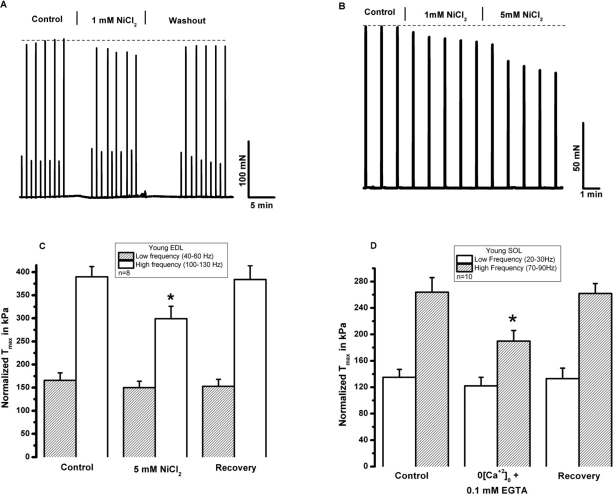
Extracellular Ni^2+^ reduces tetanic force in young skeletal muscle. **(A)** An intact EDL muscle was electrically stimulated at 37°C with low frequency and high-frequency in a bath solution with 2.5 mM [Ca]_o_ (control) and following addition of 1 mM NiCl_2_. Ni inhibits force generated with high- but not low-frequency stimulation. **(B)** An intact SOL muscle was electrically stimulated at 37°C with high-frequency in a bath solution with 2.5 mM [Ca]_o_ (control) and following addition of 1mM and 5mM NiCl_2_. A cumulative effect to NiCl_2_ is noted. (**C)** Data summary for the effects of NiCl_2_ on EDL muscle at T = 37° C (n = 8, p < 0.01). **(D)** Data summary for the effects of NiCl_2_ on SOL at T = 37° C (n = 6, p < 0.01).

**Figure 4:**
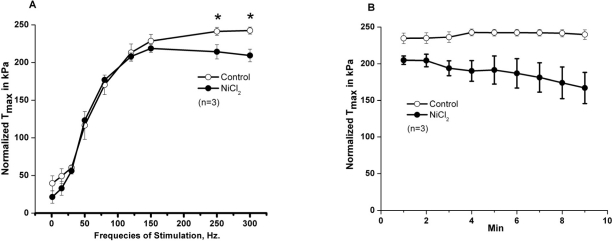
Effects of Ni^2+^ on contractility of young diaphragm muscle. **(A)** Force vs. frequency relationship in control diaphragm muscles (open circles) or diaphragm muscles treated with NiCl_2_ (solid circles). (n = 3). **(B)** Intact diaphragm muscle bundles from young mice were electrically stimulated at 37°C with high frequency (200 Hz/min) in the absence (open circles, control) or in the presence of 3 mM NiCl_2_ (solid circles, NiCl_2_).

SOCE is dependent upon extracellular calcium. To begin testing the role of extracellular Ca^2+^ in skeletal muscle contractility, Ca^2+^ was removed from the extracellular solution bathing intact, isolated extensor digitorum longus (EDL, glycolitic mostly ast-twitch) muscle fibers by chelation with 0.1 mM EGTA. Under these conditions, we observed a decrease in the contractile force following high frequency stimulation, while the low frequency component is not significantly altered (Fig. 2A). This suggests that during high frequency tetanic contractions, repetitive cycles of SR Ca^2+^ release results in loss of Ca^2+^ from intracellular SR stores to trigger entry of Ca^2+^ into muscle fibers. In multiple experiments, we observed full recovery of contractile force upon restoration of 2.5 mM [Ca^2+^]_o_ by washout of EGTA-containing solution (n=18/20) (Fig. 2B). Note that both the initial loss of force and recovery after washout are rapid, suggesting that the effect of [Ca]_o_ may be related to direct shuttling of Ca^2+^ across the sarcolemmal membrane through mechanisms that these type of experiments are not able to reveal. Our next step was to investigate if the extracellular Ca^2+^ dependence observed in EDL muscles was also present in the soleus (SOL, oxidative mostly slow-twitch) muscle. We found that removal of extracellular Ca^2+^ actually produced a larger drop in contractile force in young SOL as compared to young EDL muscles (Fig. 2C-D). Further evidence of the necessity of [Ca^2+^]_o_ entry in the maintenance of skeletal muscle contractility is provided by a series of experiments that inhibit [Ca^2+^]_o_ entry through inclusion of NiCl_2_ in the extra-cellular solution, which has been shown to specifically inhibit a component of Ca^2+^ entry in smooth muscle cells that is not affected by either nifedipine or verapamil, which suggest its selectivity to block the SOCE machinery [23, 24]. Since the experiments shown in Fig. 2 were performed at ambient temperature (~25°C) and physiological mechanisms may display dependence on temperature, we conducted these contractility experiments involving NiCl_2_ at the 37°C to establish the extent to which Ca^2+^ entry effects contraction at physiologically normal temperature (Fig. 3). These experiments revealed that a similar effect of [Ca^2+^]_o_ entry on contractility can be observed at physiological temperatures. As shown in Fig. 3A, addition of 1 mM Ni^2+^ to the extracellular solution results in a nearly instantaneous drop in the contractile force of isolated EDL muscles. The effect of Ni^2+^ was completely reversible after washout in the majority of the experiments (Fig. 3B, n=9/11). Similarly, the SOL muscles also displayed a component of contractile force that is sensitive to inhibition by NiCl_2_ (Fig. 3C). As summarized in Figs. 2D and 3D, SOL muscles demonstrate a larger Ca^2+^ dependence component in both Ca removal and SOCE blockade with Ni^2+^. As with chelation of [Ca^2+^]_o_ by EGTA we found that Ni^2+^ has more pronounced effects on the contractile force developed during high frequency stimulation than during low frequency stimulation, further suggesting that under conditions of high Ca^2+^ demand, [Ca^2+^]_o_ exerts a more significant role in skeletal muscle contractility. An intriguing observation during the NiCl_2_ experiments was the transient increase in the low frequency force (Fig. 3A). It is possible that at these lower frequencies, the addition of Cl^-^ induced Ca^2+^ release from the SR, a phenomenon previously reported by Stephenson et al [25]. This result actually provides very strong evidence that a drop in force at higher frequencies of stimulation in response to NiCl_2_ is not artifactual. From the measurement of force-frequency relationship, one can clearly see that Ni^2+^ effects were more pronounced at higher frequencies of stimulation (Fig. 4A) supporting the concept that SOCE may contribute to DPH muscle contractility under conditions of higher demand, which might have important clinical implications, since DPH fatigue can lead to serious disease and death. In Fig. 4B, DPH muscles were contracted with a high frequency of 200Hz. Clearly, a fast initial drop in force within 1min is easily detected. This initial drop in force is followed by an additional time-dependent force reduction component, since a further reduction in force is observed in the following 8min of exposure to Ni^2+^ (Fig. 4B). These data further confirm our findings in EDL and SOL muscles, and suggest that SOCE response is a universal phenomenon in different muscle types of young animals.

### Aged skeletal muscle displays reduced contractile force and negligible [Ca^2+^]_o_ dependence

**Figure 5:**
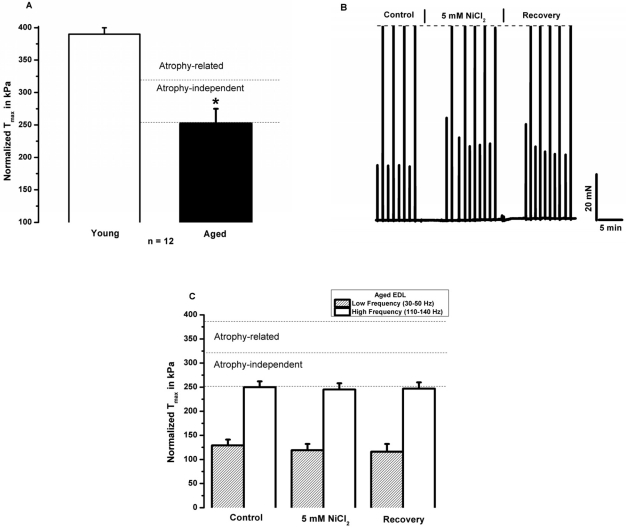
Reduced specific force in aged skeletal muscle associates with blunted [Ca^2+^]_o_ dependence. **(A)** Maximal tetanic force normalized to the cross sectional area in young (red) and aged (green) EDL muscles (n = 12, * p < 0.001). **(B)** Intact EDL muscle from aged mice subjected to the same protocol as in Figs 2-3, demonstrates that NiCl_2_ lacks an effect in aged muscle. **(C)** Summary data for the effects of NiCl_2_ and washout after treatment (n = 8). **(D)** Summary data for the effects of 0[Ca^2+^]_o_ and washout after treatment (n = 6).

Extending our contractile measurements to preparations of EDL muscles from aged mice, we first confirmed our previously determined decrease of ~30% in normalized specific contractile force in this tissue (Fig. 5A). Since this reduction in force is already normalized to the different cross-sectional areas of young and aged muscles, such difference is independent of age related atrophy. These experiments revealed quite surprising phenotypic changes in aged muscle - a remarkable suppression of the sensitivity of aged muscle to inhibition of contractile force by inhibition of [Ca^2+^]_o_ entry. As illustrated in Fig. 5B, there are no detectable effects of Ni^2+^ on the high-frequency tetanic force in aged muscles. Similarly to the young muscles, Ni^2+^ had a tendency to increase the low frequency force, but by the end of the recovery force the low frequency force returned to its control levels. Over the course of several experiments, we found only negligible effects of extracellular Ni^2+^ on muscle contractility (Fig. 5C). Additionally, we found that chelating [Ca^2+^]_o_ with EGTA had no significant acute impact on the contractile function of aged EDL skeletal muscle (Fig. 5D). Complementary experiments in aged soleus muscle demonstrated similar effects of Ni^2+^ and 0[Ca^2+^]_o_ on contractility (Fig. 5D). These results indicate that the acute functional contribution of [Ca^2+^]_o_ to skeletal muscle contractility is impaired in aged skeletal muscles, and stands in stark contrast to the significant contribution of [Ca^2+^]_o_ to acute contractility in young muscle.

### Mechanisms of loss of Ca^2+^-dependence in aged skeletal muscle

**Figure 6:**
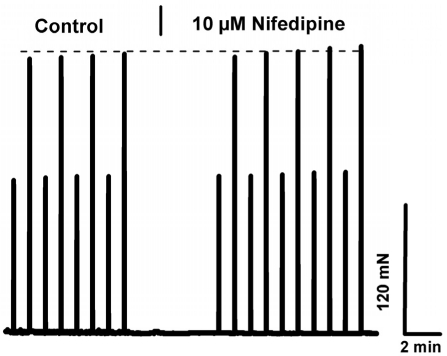
L-type/DHPR inhibition does not explain ex-tracellular Ca^+2^ dependence in young skeletal muscles. Intact EDL muscle from young mice subjected to the low/high frequency stimulation protocol demonstrates that 10 μM nifedipine lacks an effect in young muscle.

**Figure 7:**
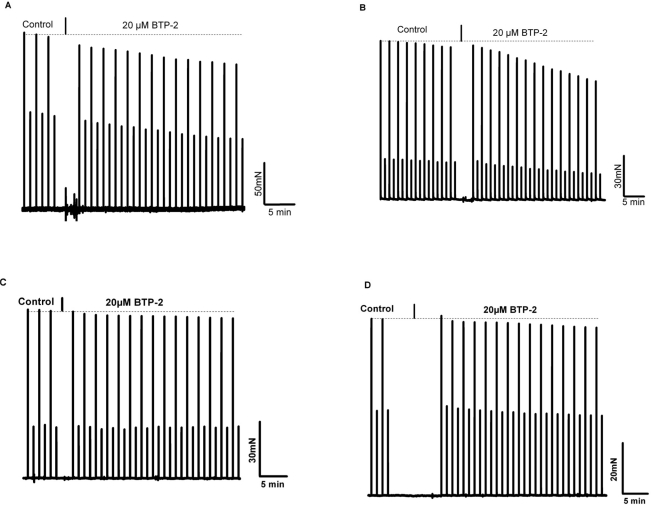
BTP-2, a selective and potent inhibitor of SOCE reduces contractile force in young but not in aged skeletal muscle. **(A)** Intact EDL muscle stimulated with low and high frequencies every minute at 37°C; in control (no drug) contractions are shown, followed by the progressive effect of 20 μM BTP-2. On average the decrease in force with 20 μM it was 19 ± 4%. **(B)** Same protocol as in panel A, but using young SOL muscles, representative of 3 experiments. In these 3 experiments force decreased by 31 ± 7% in 15 min in SOL muscles. **(C)** EDL muscles from aged Wt mice were equilibrated with 30 and 80 Hz under control condition and then were exposed to 20 μM BTP-2. **(D)** SOL muscles from aged WT mice tested with the same protocol as shown for aged EDL muscles in panel C. No statistical differences between control and BTP-2 were seen in aged EDL and SOL muscles.

Our results indicate that the dependence of skeletal muscle on [Ca^2+^]_o_ is lost in aged muscle. Elucidation of the mechanism contributing to this loss of Ca^2+^-dependence may provide a therapeutic target for increasing contractile force and function in aged skeletal muscle or perhaps new insights into the prevention of muscle loss in young subjects during aging. To establish whether the effect of [Ca^2+^]_o_ entry on contractility of young skeletal muscle was mediated by the L-type Ca^2+^ channel, we incubated the EDL or soleus muscles with nifedipine, an inhibitor of this channel, at a concentration (10 μM) previously shown to block Ca^2+^ entry via the L-type Ca channel during excitation-coupled Ca^2+^ entry (ECCE) in C2C12 myotubes [26] As shown in Fig. 6, there was no significant decrease in contractility observed with up to 13 minutes of exposure to nifedipine, a finding in agreement with previous reports from Reid et al. [27; 28]. Therefore, the mechanism that underlies [Ca^2+^]_o_ entry contribution to contractility is likely to involve pathways other than those linked to the L-type channels. Our findings further suggest that this mechanism must be functional in skeletal muscle from young, but not aged, individuals. Considering our previous findings indicate compromised SOCE in aged skeletal muscle, we hypothesize that SOCE could be one such pathway. To test this hypothesis, we examined how abrogated SOCE might affect force production in young and aged skeletal muscle. First, we examined the effects of BTP-2, which has been shown to be a specific blocker of SOCE in skeletal muscles [29], on contractility of young and aged muscle. We found that BTP-2 reduce the high frequency tetanic force produced by EDL and SOL muscles from young mice (Fig. 7A and 7B), further suggesting that a component of force generation in young, healthy skeletal muscles derives from functional SOCE. In striking contrast, BTP-2 did not have noticeable effects on the contractile force generation of aged EDL and SOL muscles (Fig. 7C and 7D).

**Figure 8:**
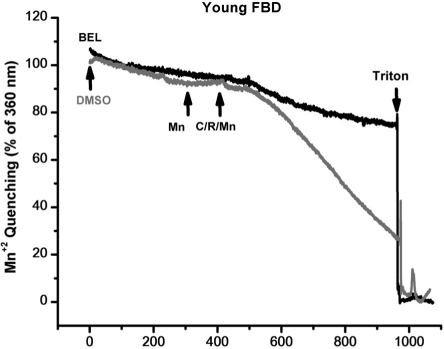
Mn^+2^ quenching of Fura-2 in FDB muscles of young muscle validates the use of BEL as an effective SOCE blocker. Enzymatically dissociated FDB muscle fibers were tested for their SOCE activity using the Mn^+2^ quenching assay. BEL was able to block most 90% of the SOCE response under these experimental conditions.

**Figure 9:**
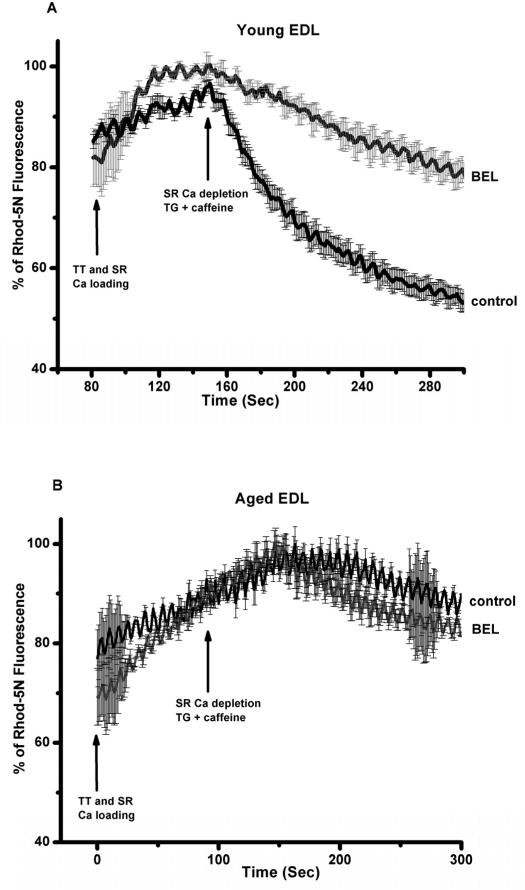
Direct visualization of SOCE in mechanically EDL skinned muscle fibers of young and old muscles with BEL reveals robust SOCE in young but compromised SOCE in aged muscle. Confocal imaging of Ca movement from the sealed T-tubules of mechanically skinned muscle fibers from young and aged mice demonstrates that SOCE is fully function in young, but severely ablated in aged muscle fibers.

**Figure 10:**
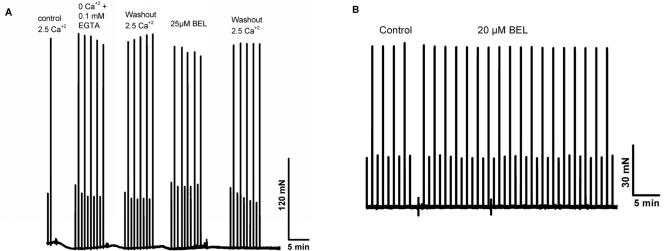
BEL reduces contractile force in young but not in aged skeletal muscle. Intact EDL and SOL muscles from young and aged muscles reveal that this agent is able to reduce the contractile force in only young muscles.

Previous studies by Bolotina and colleagues [30, 31] showed that bromoenol lactone (BEL) is a potent inhibitor of SOCE, while studies by Gong and Reid found that BEL can reduce contractile force in skeletal muscle from young adult mice [32]. Thus, a series of studies were conducted a series of experiments to test if inhibition of SOCE by BEL produced differential effects on contractility in young or aged skeletal muscle fibers. Since BEL has not been used to inhibit SOCE in primary skeletal muscle, we first validated that BEL was effective under these conditions. We conducted Ca^2+^ imaging studies in individual flexor digitorum brevis (FDB) muscle cells to measure SOCE using Mn^2+^-quenching of Fura-2 fluorescence to measure SOCE [33]. Our data with young isolated FDB muscle fibers revealed that 25 μM BEL could almost completely inhibit SOCE elicited by depletion of the SR Ca^2+^ stores using thapsigargin (TG) or caffeine/ryanodine treatment (Fig. 8). Knowing that BEL acted as a very effective blocker of SOCE in young FDB muscle cells, we then used mechanically-skinned muscle fibers to monitor Ca^2+^ exit from the T-tubules via SOCE using the fluorescence of Rhod-5N trapped in the T-tubule space as a reporter [16, 34]. Under these conditions, BEL effectively blocked SOCE as monitored with confocal imaging of Rhod-5 fluorescence in young muscle fibers (Fig. 9A), but had negligible effect in aged ones (Fig. 9B). Our last series of experiments was to investigate the effects of BEL in contractility experiments, and we found that BEL can reduce the high-frequency force production of young EDL muscle (Fig. 10A), providing additional pharmacological evidence that extracellular Ca^2+^ is linked to force production in skeletal muscle. When these experiments are conducted with aged skeletal muscle there is no decrease in contractile force following BEL application (Fig. 10B). Thus, the SOCE-dependent component of skeletal muscle contractility that is present in young skeletal muscle is no longer active in aged skeletal muscle, and parallels at least part of the loss of the contractile force in this tissue [13] (see also figures in the manuscript indicating the atrophy-related and atrophy-independent components of force generation in aged muscles and accompanying Editorial Commentary).

## DISCUSSION

As aging populations grow world-wide (It has been estimated that by 2050 more than 200 million humans will have sarcopenia), the aging-related decline in muscle function becomes a more pressing public health concern that requires more effective therapeutic approaches, which in turn requires an understanding of sarcopenia at a mechanistic level. While a portion of the decrease in muscular strength during aging can be explained by muscle atrophy, it is clear that other components contribute to reduced force production during aging. Furthermore, it is unquestionable that an array of cellular modifications [8-13] may contribute to muscle aging, and the pleiotropic nature of muscle aging muscle be recognized and accepted if we are to make significant progress towards its prevention and management.

While the role of SOCE during aging is largely unexplored, we recently had established the novel finding that SOCE is compromised in aged skeletal muscle [16], but our inaugural studies failed to directly link reduced SOCE in aged muscles with decreased contractile force in aged muscles. Now, our new data reveals that a functionally significant, SOCE-dependent component of skeletal muscle contractility in young muscle is lost in aged skeletal muscle. A viable hypothesis for atrophy-independent muscle weakness in aging, therefore, involves substantial abrogation of SOCE, a process vital to the full functionality of E-C coupling.

Our studies indicate that in young skeletal muscle, SOCE contributes to maintenance of contractile force under high frequency stimulation, where Ca^2+^ requirements may exceed the capacity of the intracellular store to provide for sustained and repetitive contractions, almost as if there is a fatigue component to the SOCE regulation of contractile force. The fact that force generation in skeletal muscle from aged animals is significantly reduced under conditions of high-frequency stimulation suggest that this tissue has lost its ability to use [Ca^2+^]_o_ during periods of increased demand, a phenomenon we recently introduced [18]. Our data now suggests that SOCE is actually required for repetitive stimulation of skeletal muscles, and its loss in aging significantly contributes to the loss of contractile function in the skeletal muscle.

Intriguingly, reduced SOCE during the aging process is not a phenomenon limited to skeletal muscles, but is also present in other tissues, such as fibroblasts and neuronal cells [35, 36]. In addition, it is clear from the recent report by Stiber, that STIM1, and hence SOCE, is required for the normal development of skeletal muscle [22]. Our findings that [Ca^2+^]_o_ contributes directly to contractility in young skeletal muscles is different than previous findings - that [Ca^2+^]_o_ does not normally contribute to skeletal muscle contractility, except in a subpopulation of aged muscle fibers [37]. This may be due to the fact that most of the earlier studies suggesting a relative lack of effect of [Ca^2+^]_o_ on muscle contractility were not performed under conditions that could allow detection of the effect that we observe, including the fact that many of these studies were performed in amphibian muscle, and also in some cases, these studies were conducted in single muscle fibers that might behave in ways that are different from the intact, anatomical muscle. Also, since Delbono and collaborators showed that a subpopulation of aged muscle fibers were dependent on extracellular calcium entry, while another subpopulation was not dependent on extracellular calcium entry, it does make sense that in a intact muscles, where all muscle fibers are combined, such difference is not observed. Furthermore, our results come from use of a previously unexploited high-frequency stimulation (using 80-200 Hz of 500-1000ms duration, with single pulses of 1ms duration) paradigm that mimics states when demand for Ca^2+^is high to elucidate the functional effects of SOCE on the contractility of three distinct intact muscle types (EDL, soleus and diaphragm) from mice. In addition, more potent SOCE inhibitors are now available and utilization of a small but effective concentration of 0.1 mM EGTA in the zero Ca^+2^ solution was critical for full observation of these effects, likely required to buffer the large amount of calcium in the T-tubular system. One of our most significant findings is that skeletal muscle contractile function under conditions of low-frequency stimulation remains mostly [Ca^2+^]_o_-independent, while significant contribution of SOCE to contractility is more prominent under high frequency stimulation. In line with our hypothesis that there is a loss of functional SOCE in aged muscle, there was no effect on force by the application of EGTA, NiCl_2_, BTP-2, and BEL in aged muscle. The utilization of *ex vivo*, intact isolated muscles offers the advantage of robust preparations, as evidenced by our previous report that LDH is essentially undetectable in the extracellular bathing solution of our preparations [38, 39]. Our findings indicate that the SOCE-dependent component of contractility we observed in young muscle is lost in aged muscle, which also display reduced SOCE activity (See Figs 8-9 and [16]). Such findings provide the possibility that increasing SOCE activity in aged muscle may restore this lost component of contractile force and increase force generation. In light of our present results and our recent work with MG29 null mice [16, 17], the parallels between defective SOCE and reduced MG29 levels in aged skeletal muscle may be of significant physiological import. Indeed, these findings suggest that modulation of MG29 function in aged skeletal muscle provides a potential therapeutic target to enhance SOCE and increase muscle contractility in aged patients. This finding is further supported by the recent association of mitsugumins in human muscle diseases [40]. Yet, another molecule of interest might be MIP/MTMR14, since we recently showed that mice lacking this molecule display phenotypic muscle changes that mirror muscle wasting and muscle weakness during aging [13].

In summary, we show for the first time that SOCE plays a significant role in normal contractility in young, healthy skeletal muscle. In striking contrast, in aged skeletal muscle, we now show that compromised SOCE is linked to decreased contractile force. We postulate that a chronically compromised SOCE leads to an equally chronic reduction in the amount of Ca^2+^ stored in the SR, creating a negative feedback loop, less Ca^2+^ is available, less is released, with less force being produced. Interestingly, the absolute force produced by young, healthy muscles when SOCE is inhibited approximates the amount of force produced by aged muscles. Thus, SOCE should be seen as an important functional biomarker of muscle function during aging, and it may provide a suitable target for therapeutic intervention for muscle diseases. Whether the loss of SOCE contributes to muscle atrophy during sarcopenia, or whether it is the reflection of the pleiotropic adaptive response during aging, is still very much an open question.

## MATERIALS AND METHODS

### Muscle Preparation

[17, 34, 41]. Intact extensor digitorum longus (EDL), diaphragm (DPH) and soleus (SOL) muscles of wild type (Wt) male mice, 5 and 24 month old, were surgically removed from tendon to tendon by blunt dissection and immediately placed in a dissecting dish containing a modified bicarbonate Ringer solution with the following compositions (mM). In the case of the DPH, small bundles were prepared in such a way that they could be mounted from the rib cage and the central tendon. The pH was adjusted to between 7.4 with NaHCO_3_, followed by the addition of fetal bovine serum (to 0.2%) to increase viability of the dissected muscle. The solution was continuously aerated with a gas mixture consisting of 95% O_2_ and 5% CO_2_. EDL and SOL muscles were mounted vertically between two Radnoti (Monrovea, CA, USA) stimulating platinum electrodes and immersed in a 20 mL bathing chamber containing the incubation medium. The muscles were suspended from movable isometric force transducers above the chambers via the tendons, and secured to the base of the tissue support within the chambers. The analog output of the force transducer was digitized, stored and analyzed with PowerLab Software (Colorado Springs, CO, USA). For each muscle, the resting tension and the stimulatory voltage were provided by a Grass S8800 digital stimulator (West Warwick, RI, USA) and both parameters were adjusted to produce a maximal isometric tetanic force (*T_max_*).

**Table:** 

	NaCl	KCl	CaCl_2_	NaH_2_PO_4_	MgCl_2_	Glucose	EGTA
Ca	135	5	2.5	0.4	0.5	10	—
0 Ca	135	5	—	0.4	0.5	10	0.1

### Intact Muscle Protocol

After initial *T_max_* determination, the muscles were subjected to a protocol that mimics normal muscle activity (No fatigue, stimulation trains triggered at every minute, duty cycle of 2%). Muscles were placed in 2.5 mM Ca or zero Ca^2+^ and/or 5 mM NiCl_2_, 1 mm NiCl_2_, 10 μM nifedipine, or 25 μM bromoenol lactone (BEL). The intact muscles were allowed a 30-minute equilibration, during which time they were stimulated with pairs of alternating high (that produced *T_max_*) and low (that produced 1/2 *T_max_*) frequency pulse-trains administered with a periodicity of 1 minute, before any pharmacological intervention was attempted. The results indicate the relative contributions from the contractile proteins (*T_max_*) and from the SR (1/2 *T_max_*) to the stimulation [41].

### Intracellular Ca^2+^ Measurement and Mn^2+^ Quenching

[33]. For quantitative measurements of intracellular Ca^2+^], FDB muscle fibers were enzymatically isolated in a 0 Ca^2+^ Tyrode solution containing 2 mg/mL type I collagenase for 2 hours in a shaking bath at 37 °C, before being transferred to a 0 Ca^2+^ Tyrode solution without collagenase and gently triturated with a pipette. The fibers were then loaded with 5 *μ*M Fura-2-AM for 40 minutes, after which the Fura-2 AM was washed off. As fiber motion artifacts are associated with intracellular Ca release, 20 μM *N*-benzyl-*p-*toluene sulfonamide (Sigma, St. Louis, MO), a specific myosin II inhibitor, was then applied for 20 minutes. A dual-wavelength (excitation at 340 nm and 380 nm) PTI spectrofluorometer (Photon Technology International, Birmingham, NJ) was used to determine the kinetic changes of caffeine and ryanodine (C/R)-induced intracellular Ca^2+^ transients.

Mn^2+^ quenching experiment were performed as previously described with minor modifications. Briefly, FDB fibers were loaded with Fura-2/AM fluorescent Ca^2+^ indicator [16]. SR Ca^2+^ depletion with C/R (with and without 25 μM BEL) was followed by the addition of 0.5 mM MnCl_2_ (with and without 25 μM BEL) to the extracellular solution. The Mn^2+^ quenching of Fura-2 fluorescence was measured at the Ca^2+^-independent wavelength of Fura-2AM excitation (360 nm). The decay of Fura-2AM fluorescence upon Mn^2+^ addition was expressed as percent decrease in Fura-2 fluorescence per unit time.

### Confocal Imaging of Store-operated calcium entry in mechanically-skinned muscle skinned muscle fiber

These studies followed our own protocols, which are based on our original and first ever methodology to directly monitor the spatial and temporal resolution of SOCE in mammalian skeletal muscle fibers [16, 34]. Briefly, intact muscle fibers are carefully skinned in the present of 0.5mM CaCl_2_ and Rhod-5N, forcing the trapping of the dye and Ca^+2^ in the re-sealed T-tubules. Using IDL driven sub-routines, areas of interest comprising either the T-tubules or background areas can be drawn and the relative fluorescence upon activation of SOCE can be quantified. A minimum of 6 ROIs from each muscle fiber from 9 muscle fibers from 3 mice was employed to obtain the average SOCE activity in young and aged mice.
